# Erythrocyte-Derived Nanoparticles with Folate Functionalization for Near Infrared Pulsed Laser-Mediated Photo-Chemotherapy of Tumors

**DOI:** 10.3390/ijms231810295

**Published:** 2022-09-07

**Authors:** Jenny T. Mac, Raviraj Vankayala, Chi-Hua Lee, Bahman Anvari

**Affiliations:** 1 Department of Biochemistry, University of California, Riverside, CA 92521, USA; 2 Radoptics, Limited Liability Corporation, 1002 Health Sciences Road East, Suite P214, Irvine, CA 92612, USA; 3 Department of Bioengineering, University of California, Riverside, CA 92521, USA

**Keywords:** biomimetics, cancer, drug delivery, laser therapy, nanotechnology, photothermal therapy, red blood cells

## Abstract

Despite its common side effects and varying degrees of therapeutic success, chemotherapy remains the gold standard method for treatment of cancer. Towards developing a new therapeutic approach, we have engineered nanoparticles derived from erythrocytes that contain indocyanine green as a photo-activated agent that enables near infrared photothermal heating, and doxorubicin hydrochloride (DOX) as a chemotherapeutic drug. We hypothesize that milliseconds pulsed laser irradiation results in rapid heating and photo-triggered release of DOX, providing a dual photo-chemo therapeutic mechanism for tumor destruction. Additionally, the surface of the nanoparticles is functionalized with folate to target the folate receptor-α on tumor cells to further enhance the therapeutic efficacy. Using non-contract infrared radiometry and absorption spectroscopy, we have characterized the photothermal response and photostability of the nanoparticles to pulsed laser irradiation. Our in vitro studies show that these nanoparticles can mediate photo-chemo killing of SKOV3 ovarian cancer cells when activated by pulsed laser irradiation. We further demonstrate that this dual photo-chemo therapeutic approach is effective in reducing the volume of tumor implants in mice and elicits an apoptotic response. This treatment modality presents a promising approach in destruction of small tumor nodules.

## 1. Introduction

**Scheme 1:**
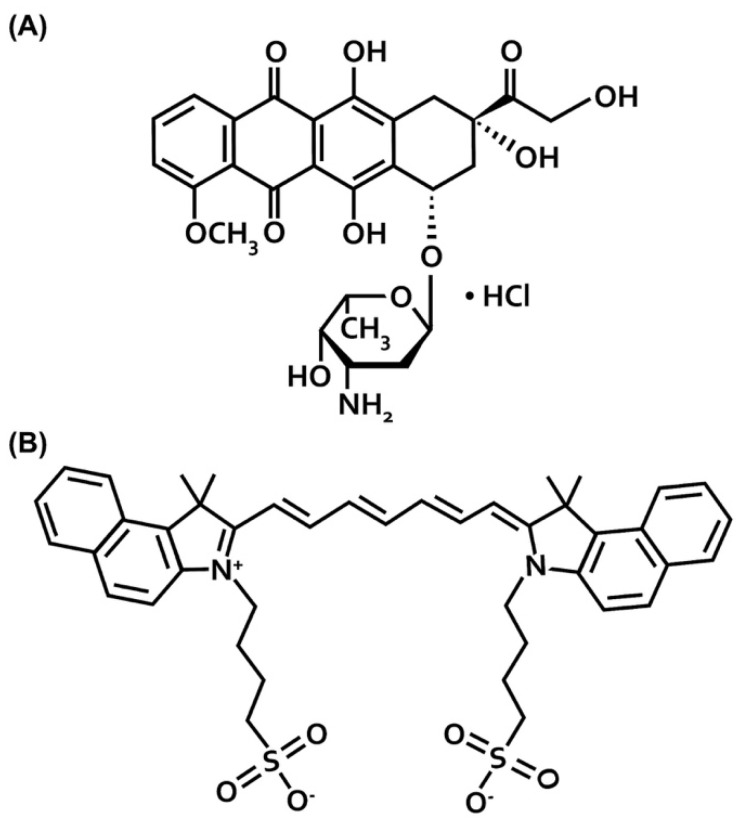
Molecular structures of (**A**) DOX and (**B**) ICG.

Despite its non-specific toxicity and side effects, chemotherapy remains the gold standard method for treatment of solid tumors [1,2]. For example, doxorubicin hydrochloride (DOX) (Scheme 1A), an anthracycline topoisomerase inhibitor, is a highly effective anti-neoplastic agent. The PEGylated liposomal doxorubicin nanoparticle formulation (DOXIL^®^) has been approved by United Stated Food and Drug Administration (FDA) for the treatment of ovarian cancer patients with relapse [3,4,5]. However, severe toxic side-effects, such as cardiotoxicity, alopecia, vomiting, leucopenia, and stomatitis, have hindered the successful use of DOX [6,7,8].

One strategy for reducing the side effects and systemic toxicity of DOX and other chemo-drugs while improving therapeutic efficacy is to combine chemotherapy with other treatment modalities. Nanoparticle-based anticancer drug delivery promises a technology to accommodate multiple drug molecules or therapeutic modalities and achieve synergistic effects [9]. Nanoparticles responding to external stimulations (e.g., light, magnetic field, ultrasound) or internal stimulations (e.g., reduction/oxidation, pH, and enzymatic activity) have been developed with demonstrated efficacies [10,11]. These stimuli can provide excellent spatiotemporal and dosage control for drug release in tumor tissues with reduced systemic toxicity.

Photothermal therapy (PTT), which is based on transduction of the absorbed light energy to generate heat, presents a particular external stimulation approach for killing cancer cells. Near infrared (NIR) laser-mediated thermal therapy has been investigated to induce release of drug molecules [12,13,14]. To date, indocyanine green (ICG) (Scheme 1B) remains the only FDA-approved NIR-activated agent for clinical applications ranging from ophthalmic angiography, cardiocirculatory measurements, assessment of hepatic function, and blood flow evaluation. ICG has also been used as an agent to mediate PTT [15]. However, the drawbacks of ICG include its short half-life within plasma (~2–4 min), with nearly exclusive uptake by hepatocytes and elimination through the hepatobiliary mechanism and non-specificity.

Encapsulation of ICG and DOX into a nano-sized delivery system can provide a method to extend the circulation times of both the agents, so that higher amounts of the payloads can be accumulated at specific target sites [16,17]. Combined with molecular targeting, therapeutic efficacy can be further enhanced. For example, a particularly promising target for ovarian cancer is the folate receptor-α (FRα), which is over-expressed in both primary tumor tissue and in metastatic tumor deposits [18,19,20,21].

Erythrocyte-derived platforms are gaining increased attending for delivery of various biomedical cargos [22,23,24,25,26]. Our group reported the first demonstration of nano-sized particles derived from red blood cells (RBCs) containing ICG for NIR fluorescence imaging and photothermal destruction of cells [27]. We refer to these constructs as NIR erythrocyte-derived transducers (NETs).

In this study, we have engineered erythrocyte-derived nanoparticles that encapsulate both ICG and DOX. Additionally, the surface of these particles is functionalized with folate to target FRα. We refer to these particles as folate-functionalized ICG + DOX containing NETs (F-IDNETs). To the best of our knowledge, herein, we demonstrate for the first time that, when activated by pulsed (0.5 s) NIR (808 nm) laser irradiation, these particles mediate photothermal effects and induce the release of DOX. The significance of pulsed laser irradiation, as compared to continuous wave (CW) irradiation, which can be on the order of minutes, is that heat generation can remain confined to the target site to avoid non-specific thermal damage to other tissue sites. Additionally, pulsed laser irradiation substantially reduces the operating time for tumor removal during surgical procedures. Using xenograft ovarian tumor cells implanted subcutaneously in mice, we also demonstrate that F-IDNETs in conjunction with 808 nm pulsed laser irradiation elicit a greater apoptotic response of the tumor and induce a higher reduction in tumor volume as compared to F-NETs (folate-functionalized NETs without DOX) and the non-encapsulated forms of ICG, DOX, and the ICG + DOX complex.

## 2. Results

### 2.1. Physical and Optical Characteristics

**Figure 1:**
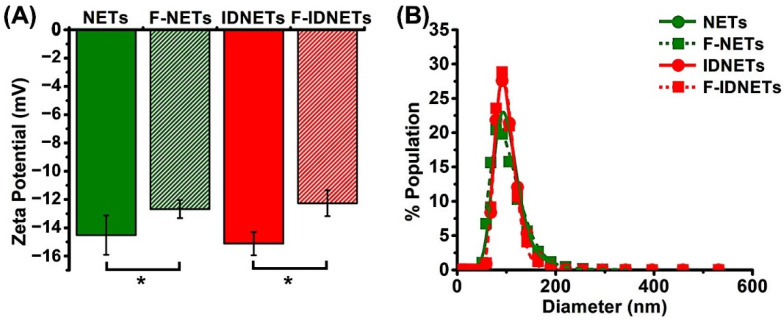
(**A**) Zeta potentials and (**B**) hydrodynamic diameter distributions, as measured by DLS (symbols) and the lognormal fits to the measurements (traces) for the various sets of erythrocyte-derived nanoparticles. Particles were suspended in isotonic PBS during measurements. In panel (**A**), each bar represents the mean value of three independent measurements, and error bars are the SD values from the mean. Asterisk (*) indicates a statistically significant difference between the mean values for the indicated pairs at *p* < 0.05 (two-tailed *t*-test).

The mean ± standard deviation (SD) values of the zeta potentials of NETs, F-NETs, IDNETs (ICG + DOX containing NETs), and F-IDNETs measured in isotonic (1X) phosphate buffer saline (PBS) were −14.52 ± 1.39, −12.68 ± 0.64, −15.12 ± 0.82, and −12.26 ± 0.92 mV, respectively (Figure 1A). The negative values of the zeta potentials can be attributed to the carboxyl groups of sialoglycoproteins on RBCs [28]. Folate functionalization, resulted in a small but statistically significant difference (*p* < 0.05) in the mean zeta potential of F-NETs and F-IDNETs compared to their non-functionalized counterparts. This difference may be due to possible camouflaging of some of the carboxyl groups by the 1,2-distearoyl-sn-glycero-3-phosphoethanolamine (DSPE)-polyethylene glycol (PEG)-folate used in the functionalization process.

The various sets of the nanoparticles had similar hydrodynamic diameter distributions, as measured by dynamic light scattering (DLS) (Figure 1B). The mean peak values of the hydrodynamic diameters of NETs, F-NETs, IDNETs, and F-IDNETs, as determined by fitting lognormal functions to the measured distribution profiles, were 92.98, 87.38, 91.15, and 93.01 nm, respectively (Figure 1B).

**Figure 2:**
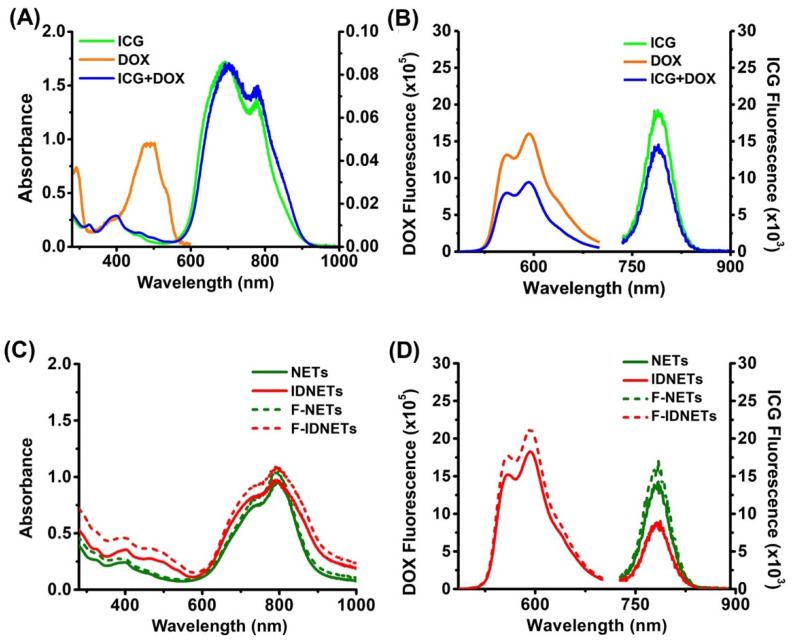
Optical spectra. (**A**) Absorption and (**B**) fluorescence emission spectra of free ICG (17.3 µM), DOX (2.6 µM), and ICG (17.6 µM) + DOX (2.6 µM) in isotonic PBS. (**C**) Absorption and (**D**) fluorescence of NETs, IDNETs, F-NETs, and F-IDNETs. In panel (**A**), the right ordinate corresponds to absorbance values of DOX and the left ordinate corresponds to absorbance values of ICG and ICG + DOX. In panels (**B**,**D**), the respective left and right ordinates correspond to the fluorescence emission intensities associated with DOX and ICG. Fluorescence emission spectra were obtained in response to 470 and 720 nm for DOX and ICG, respectively.

The absorption spectra of free (non-encapsulated) ICG (17.3 μM), DOX (2.6 μM) and ICG (17.6 µM) + DOX (2.6 µM) dissolved in 1X PBS are shown in Figure 2A. The spectral peaks of free ICG at 690 and 776 nm correspond to H-like aggregate and monomeric forms of ICG, respectively [29]. DOX has relatively small absorbance values in the Ultraviolet-Visible (UV-Vis) range, consistent with previously reported spectra by other investigators [30]. Upon mixing the ICG and DOX together, ICG spectral peaks are red-shifted to 700 and 782 nm, consistent with the formation of J-like aggregates. In such aggregates, there is a head-to-tail arrangement of the transition dipole moments of the chromophore, where the excited state is split into two new non-degenerate states with different energy levels [29,31]. For J-aggregates, only transitions to the lower level of the split excited state are allowed, causing a bathochromic (red) spectral shift. The fluorescence emission spectrum of free DOX in response to 470 nm excitation shows spectral peaks at 558 and 592 nm (Figure 2B), similar to those reported in literature [30]. Formation of aggregates of ICG + DOX provides a mechanism for reduced fluorescence emission of these aggregates, as compared to the emission of free DOX.

As compared to free ICG, the highest absorbance value of ICG when encapsulated into the RBC-derived nano-constructs was at 800 nm (Figure 2C). This result indicates that the fraction of the monomeric form of ICG was higher than the H-like aggregate fraction in these constructs. The bathochromic spectral shift in the monomeric absorption of free ICG from 776 to 800 nm when encapsulated is consistent with our previous results [27]. This shift can be attributed to the binding of ICG molecules to phospholipids and membrane proteins of the RBC-based constructs, causing a change in electronic energy levels of ICG, similar to the mechanism underlying the formation of J-aggregates. Presence of a near constant absorption shoulder in the range of 480–515 nm for IDNETs and F-IDNETs is indicative of successful encapsulation of DOX in these non-constructs.

Fluorescence emission spectra of the nano-constructs further demonstrate the successful encapsulation of both ICG and DOX (Figure 2D). In response to 470 nm excitation, there was fluorescence emission in the visible spectrum from IDNETs and F-IDNTs (Figure 2D), which was similar to the spectrum of free DOX (Figure 2B). Similarly, the NIR emission spectrum resulting from photoexcitation of ICG in the nanoparticles at 720 nm (Figure 2D) resembled the spectrum of free ICG (Figure 2B). Consistent with the reduced NIR emission intensity of non-encapsulated ICG + DOX, the NIR emission intensity of IDNETs and F-IDNETs was also reduced, which can be attributed to the aggregation-induced quenching resulting from the interaction between ICG and DOX.

### 2.2. Photothermal Response and Photostability of Nanoparticles

**Figure 3:**
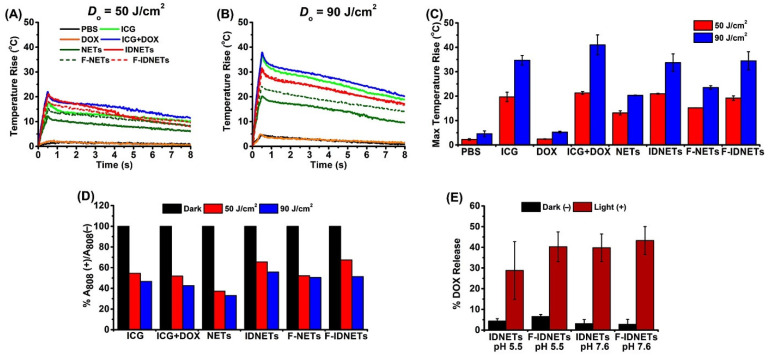
Photothermal response of nanoparticles and non-encapsulated materials and DOX release quantification. Representative radiometrically-measured changes in temperature rise of various agents in isotonic PBS in response to 808 nm pulsed (0.5 s) laser irradiation at radiant exposures of (**A**) *D*_o_ = 50 J/cm^2^ and (**B**) *D*_o_ = 90 J/cm^2^ are shown. Legends in panel (**A**) also correspond to traces shown in panel (**B**). (**C**) Average values of the maximum temperature rise. (**D**) Percentage of ICG absorbance value retained following irradiation. (**E**) Mean percentage of DOX released from IDNETs and F-IDNETs in the dark (without laser irradiation) in response to acidic pH 5.5 and in response to 0.5 s laser irradiation at *D*_o_ = 25 J/cm^2^ in nearly neutral pH 7.6. Error bars in panels (**C**,**E**) represent SDs from the mean.

Representative photothermal response of the nanoparticles and non-encapsulated agents (control samples) to pulsed (0.5 s) 808 nm laser irradiation at radiant exposure (fluence) *D*_o_ = 50 and 90 J/cm^2^, measured by non-contact radiometry, are shown in Figure 3A–C. Free DOX (2.6 µM) exhibited a slight temperature increase (e.g., ~5 °C) in response to *D*_o_ = 90 J/cm^2^), since it has minimal absorption at 808 nm (Figure 2A), and its photothermal response was similar to that of PBS (Figure 3A,B). F-IDNETs and IDNETs, fabricated using 774 µM ICG in the loading buffer, had similar photothermal responses when irradiated at *D*_o_ = 50 J/cm^2^ and 90 J/cm^2^ (e.g., maximum temperature rise of ~35 °C for both samples in response to *D*_o_ = 90 J/cm^2^), confirming that folate did not make a contribution to the photothermal response. The lower temperature rise associated with NETs and F-NETs (fabricated using 1.1 mM ICG in the loading buffer) may have been due to their lower number density in solution as compared to F- IDNETs and IDNETs. As expected, increasing *D*_o_ from 50 to 90 J/cm^2^ resulted in higher peak temperature rises (Figure 3C).

**Figure 4:**
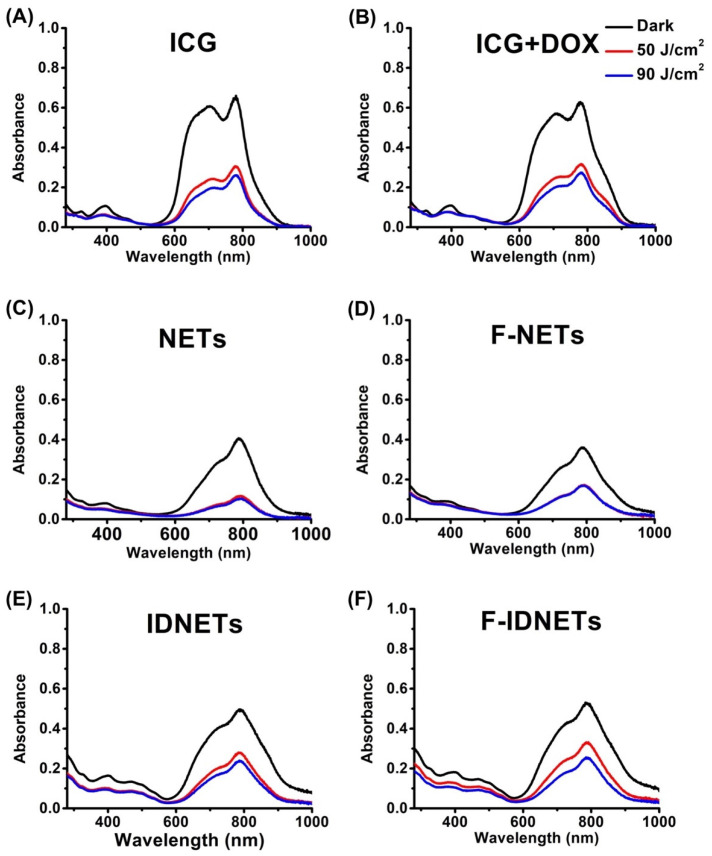
Effects of pulsed laser irradiation on non-encapsulated (free) and encapsulated ICG absorption. Absorption spectra of (**A**) free ICG, (**B**) free ICG + free DOX, (**C**) NETs, (**D**) F-NETs, (**E**) IDNETs, and (**F**) F-IDNETs in the dark (without laser irradiation) and immediately after pulsed (0.5 s) 808 nm laser irradiation at radiant exposures of *D*_o_ 50 and 90 J/cm^2^. All agents were in isotonic PBS during laser irradiation and spectral recordings.

We obtained the absorption spectra of the samples following laser irradiation at *D*_o_ = 50 and 90 J/cm^2^ (Figure 4). All samples showed reduced absorbance values in the range of 600–900 nm, indicative of photo-degradation of ICG. In particular, there were ~30–60% reductions in absorbance values at 808 nm (Figure 3D). These results are consistent with our previous findings that indicated photo-degradation of ICG in response to CW laser irradiation [27].

Effects of pH and laser irradiation-induced release of DOX from IDNETs and F-IDNETs are shown in Figure 3E. Incubation of IDNETs and F-IDNETs at pH 5.5 (representative of the acidic conditions of lysosomes and tumor microenvironment [32]) in the dark and without laser irradiation resulted in ~4.3 ± 1.2 and 6.6 ± 0.8% of DOX, respectively. These leakage values were similar to the respective values of 3.1 ± 2.0 and 2.8 ± 2.4% at pH 7.6, indicating that the acidic environment did not provide a sufficient mechanism for the release of DOX. In response to pulsed 808 nm laser irradiation at *D*_o_ = 25 J/cm^2^, the respective levels of DOX released from IDNETs and F-IDNETs were 28.8 ± 13.9 and 40.3 ± 7.2% at pH 5.5 and 39.8 ± 6.7 and 43.3 ± 6.8% at pH 7.6. We attribute the release mechanism to photothermally-induced structural changes to the membrane of the particles to mediate the release of DOX. Such structural changes have been previously reported for ICG-encapsulating polymeric material when laser-heated to temperatures >40 °C [33]. With increased radiant exposure levels, it is possible that the particles would burst. A key finding from these experiments was that laser irradiation had a much greater effect in release of DOX from both IDNETs and F-IDNETs, as compared to the low pH (5.5).

### 2.3. In Vitro Photothermal-Chemo Effects

**Figure 5:**
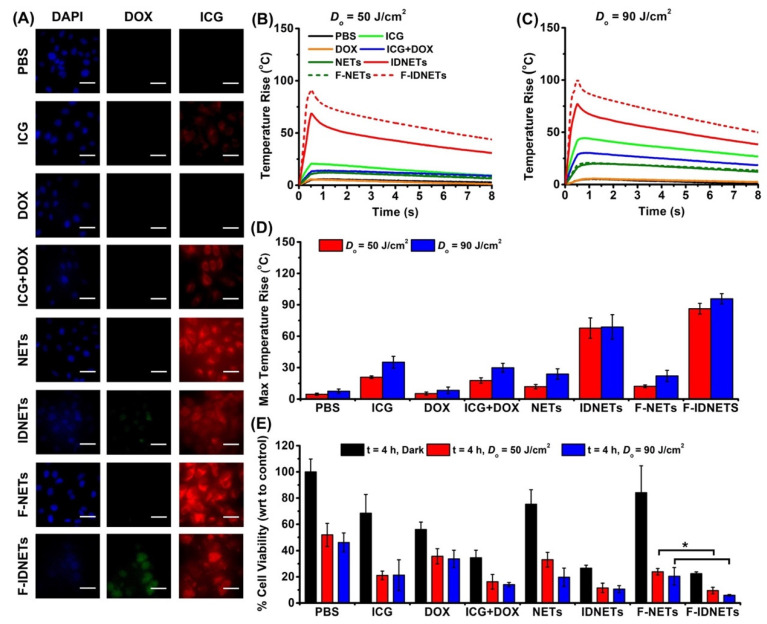
In vitro results. (**A**) Falsely colored fluorescence images of SKOV3 ovarian cancer cells after 4 h of incubation with various agents. Blue: DAPI, Green: DOX, and Red: ICG. Scale bars correspond to 50 μm. Panels (**B**,**C**) present illustrative radiometrically-measured changes in the temperature of SKOV 3 cells suspensions in response to 808 nm pulsed (0.5 s) laser irradiation at radiant exposures of (**B**) *D*_o_ = 50 J/cm^2^ and (**C**) *D*_o_ = 90 J/cm^2^ following incubation with various agents. (**D**) Average values of the maximum temperature rise of SKOV3 cells. (**E**) Average fraction of cells remaining viable following laser irradiation, with respect to (wrt) control (non-irradiated cells). Error bars in panels (**D**,**E**) represent standard deviation values from the mean. Single asterisks (*) indicate statistically significant differences between the indicated pairs (*p* < 0.05) (one-tailed *t*-test).

Fluorescence images of SKOV3 ovarian cancer cells after 4 h of incubation with various agents are shown in Figure 5A. Greater NIR emission (red channel), indicative of higher uptake, were observed using the nanoparticles as compared to free agents. Higher DOX fluorescence observed in SKOV3 cells incubated with F-IDNETs as compared to IDNETs can be attributed to a greater degree of specific targeting provided by folate functionalization of the particles.

Representative photothermal response of SKOV3 cells to pulsed 808 nm laser irradiation at *D*_o_ = 50 J/cm^2^ and 90 J/cm^2^, following incubation with the various agents, are shown in Figure 5B–D. The greatest radiometric surface temperature rise was observed from cells incubated with F-IDNETs (Figure 5B,C). These results suggest that F-IDNETs offered the greatest specificity to achieve the highest uptake level by the SKOV3 cells, as compared to other agents. The lack of a linear increase in maximum temperature rise (Figure 5D) for IDNETs and F-IDNETs when increasing *D*_o_ from 50 to 90 J/cm^2^ may be due to variations in the number density of the particles in different irradiation experiments. The higher temperature profiles due to incubation with free ICG (Figure 5B–D), as compared to NETs and F-NETs, may have resulted from a relatively low number concentration of the particles, giving rise to a lower effective absorption.

The greatest fraction of laser-induced cell death was in response to incubation with F-IDNETs (Figure 5E). There was a statistically significant reduction in cell viability following incubation with F-IDNETs, as compared to IDNETs at both radiant exposure levels of *D*_o_ = 50 J/cm^2^ and 90 J/cm^2^, suggesting that functionalization with folate resulted in higher uptake of the particles and, subsequently, greater photo-destruction. There was also cell death following incubation with F-IDNETs, IDNETs, ICG + DOX, and DOX in the dark (without laser irradiation), indicating the role of DOX as a cytotoxic agent to induce cellular death. We have previously reported that RBC-based nanoparticles (i.e., NETs) are internalized by cancer cells and localized to the lysosomes [34]. The acidic environment of the lysosomes [35] (pH in the range of 4.5–5.5) can provide the mechanism for the lysis of the particles and release of DOX in the absence of laser irradiation. However, laser irradiation increased the fraction of dead cells to confirm the synergistic effects of chemotherapy and PTT.

### 2.4. In Vivo Photothermal-Chemo Effects

**Figure 6:**
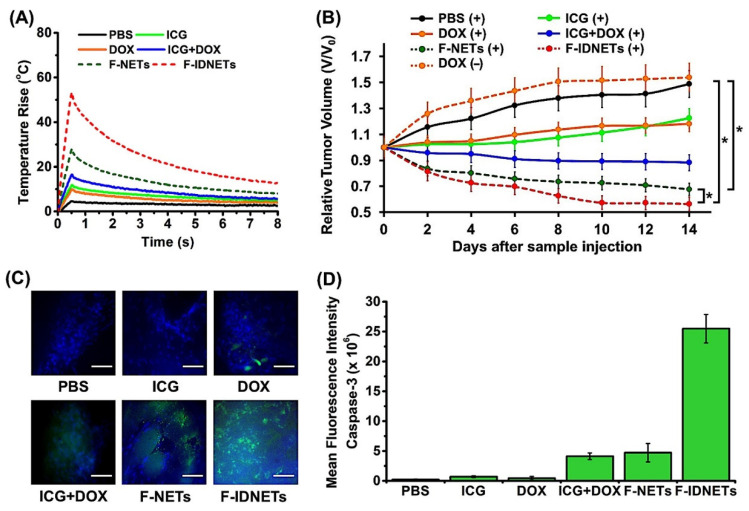
In vivo results. (**A**) Representative radiometrically-measured changes in skin surface temperature of intact mice with subcutaneous tumors in response to 808 nm pulsed (0.5 s) laser irradiation at radiant exposure of *D*_o_ = 90 J/cm^2^ performed at 24 h after i.v. administration of various agents. (**B**) Mean values of changes in tumor volumes for various treatments relative to initial tumor volume (V_0_) at day zero (just prior to administration of agents). (+) and (−) signs indicate with and without laser irradiation, respectively. Error bars are SDs from the mean (n = 6 animals per treatment). (**C**) Fluorescence images of sectioned tumors obtained by immunohistochemical staining using FITC-labeled caspase-3 antibody obtained within 24 h after laser irradiation in a subset of animals (scale bars = 50 µm). (**D**) Spatially averaged fluorescence emission intensity associated with caspase-3 corresponding to the images shown in panel (**C**) after background subtraction. Single asterisks (*) indicate a statistically significant difference (*p* < 0.05) between the indicated pairs in panels (**B**,**D**) (one-tailed *t*-test). Error bars in panels (**B**,**D**) represent SDs from the mean.

We performed in vivo experiments to evaluate the efficacy of combined photothermal and chemo effects on the destruction of SKOV3 ovarian xenograft tumors implanted subcutaneously in Nu/J nude mice. Pulsed (0.5 s) laser irradiation (808 nm) was done 24 h after intravenous (i.v.) injection of various agents via the tail vein. Illustrative in vivo radiometric surface temperature profiles in response to laser irradiation at *D*_o_ = 90 J/cm^2^ are shown in Figure 6A. Greatest temperature rise (~53 °C) was observed in response to laser irradiation in conjunction with the administration of F-IDNETs. The smaller temperature rise associated with F-NETs may have arisen from the lower absorbance value for these particles, either to lower ICG loading efficiency and/or lower number density of the particles in solution. The lower temperature profiles associated with non-encapsulated agents (ICG, DOX, and ICG + DOX ) suggests that these materials accumulated at lower quantities within the tumor sites.

There were ~20% reductions in tumor volumes in response to the administration of F-NETs and F-IDNETs, as early as two days post laser irradiation. By 14 days, there was a significantly greater reduction in tumor volume (~45%), resulting from i.v. injection of F-IDNETs and pulsed laser irradiation, as compared to ~30% reduction due to F-NETs (*p* < 0.05) (Figure 6B). There was only about 10% reduction in tumor volume in response to ICG + DOX injection in conjunction with laser irradiation. Administration of free DOX (with or without laser irradiation), or free ICG in conjunction with laser irradiation, did not present an effective treatment approach as the tumors continued to grow, suggesting that these agents did not accumulate in the tumors at sufficient levels. The combination of chemotherapy and PTT mediated by F-IDNETs resulted in significant delayed tumor growth, whereas ICG-mediated PTT or chemotherapy alone did not inhibit tumor growth.

**Figure 7:**
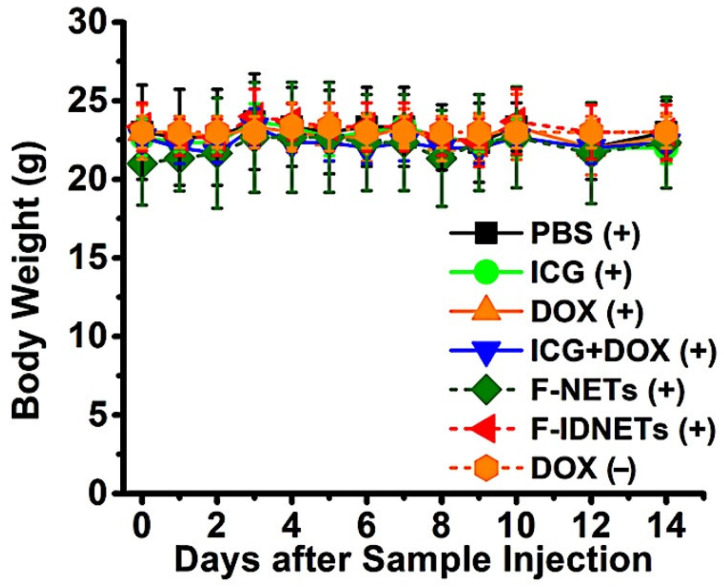
Average body weights of mice following various treatments. Measurements were obtained every day during week 1 and every other day during week 2. (+) and (−) signs indicate with and without laser irradiation, respectively. Error bars represent SDs from the mean. n = 6 mice per each treatment.

To investigate the presence of cellular apoptosis in response to the injection of various agents in conjunction with pulsed 808 nm laser irradiation, some of the tumors were extracted immediately after laser irradiation and imaged by fluorescence immunostaining to detect the presence of caspase-3. The greatest green fluorescence due to fluorescein isothiocyanate (FITC) staining was observed from the tumors of the animals treated with F-IDNETs in conjunction with laser irradiation (Figure 6C), indicative of the highest level of caspase-3 activation in these animals. Our quantitative analysis of the images revealed a significantly higher level of caspase-3 associated fluorescence emission in mice treated with F-IDNETs and laser irradiation, as compared to those with F-NETs and laser irradiation (*p* < 0.05) (Figure 6D). The mice body weights remained nearly constant in all animals during the 14 days survival time post laser irradiation (Figure 7).

## 3. Discussion

In this study, we have engineered nanoparticles derived from erythrocytes that contain two FDA-approved agents: ICG as the photo-activated agent, which enables NIR photothermal heating, and DOX as a chemotherapeutic agent to further enhance therapeutic efficacy. Additionally, the surface of the nanoparticles is functionalized with folate to target the folate receptor-α, which is particularly over-expressed in epithelial ovarian cancer cells [36]. While we have focused on functionalization of these nanoparticles with folate, they can also be functionalized with antibodies [37] to expand the repertoire of the receptors that could potentially be targeted.

Our in vitro studies using ovarian cancer cells and in vivo studies based on tumor xenografts in mice support our hypothesis that the dual treatment modality based on photothermal heating and photo-induced release of DOX combined with folate targeting can provide a more effective approach in killing the cancer cells as compared to single treatment modality using photothermal heating or chemotherapy (Figure 5E and Figure 6B). The apoptotic response resulting from F-IDNETs in conjunction with laser irradiation (Figure 6C,D) suggests that photo-induced damage to the membrane of lysosomes, the localization site of erythrocyte-derived nanoparticles [34], can provide the basis for apoptosis by allowing the release of specific enzymes (e.g., cathepsin proteases) into the cytosol to activate pro-apoptotic protein mediators [38]. The higher apoptotic activity resulting from F-IDNETs can be attributed to the presence of DOX in F-IDNETs [39].

The laser pulse duration (∆*τ*_laser_) determines the spatial confinement of heat during irradiation time. Assuming a spherically-shaped tumor nodule, its thermal relaxation time (∆*τ*_relaxation_) can be expressed as [40]:

(1) $$∆\tau_{\mathrm{relaxation}}=\frac{d^{2}}{24\alpha },$$

where *d* is the diameter and α is the thermal diffusivity. For pulse durations less than the thermal relaxation time of the target tumor nodule, heat diffusion away from the target site is minimal during the laser pulse. As a first order of approximation, assuming ∆τ_laser_ = ∆τ_relaxation_ and α = 1.1 × 10^−7^ m^2^/s (for water), the laser pulse duration of 0.5 s is sufficient for thermal confinement within a targeted tumor nodule with d ~1.15 mm. In the case of ovarian cancer, which often metastasizes to intraperitoneal sites, tumor nodules with diameters of ~≤1 mm are formed along critical anatomical structures. Such tumors can be difficult to remove surgically, if not impossible. To achieve optimal cytoreduction, extensive surgical procedures, such as diaphragm and gastric stripping, splenctomy, distal pancreatectomy, or tumor resection from the porta hepatis, may be required. Laser irradiation can serve as a viable therapeutic option in such cases. For destruction of such tumors, short laser pulses of appropriate duration (e.g., ∆τ_laser_ ≤ 0.38 s for a 1 mm tumor nodule) will be needed to induce localized hearing without imparting thermal injury to surrounding normal tissues. The rapid and spatially localized pulsed laser irradiation should reduce surgical complications and blood loss.

Another advantage of this dual treatment approach is that lower doses of the chemotherapeutic drug may be administered since the photothermal mechanism contributes to tumor destruction. The administered dose of DOX in this study was 0.76 mg/kg weight of the mouse. Scaling up this dose to a human with an average weight of 75 kg, the administered dose would be 57 mg, which is lower than the dose range of 80–120 mg used in treating patients [41].

## 4. Materials and Methods

### 4.1. Fabrication of Nanoparticles

Erythrocytes were isolated from whole human blood (Biological Speciality Corporation, Colmar, PA, USA) and washed using ~310 mOsm (isotonic) PBS (referred to as the 1X PBS solution) (Fisher Scientific, Hampton, NH, USA) at ~4200× *g* at 4 °C for 10 min. Packed erythrocytes were subject to hypotonic (~80 mOsm, 0.25X PBS) treatment (~46,000× *g* at 4 °C for 20 min) to deplete the hemoglobin content of the cell, resulting in the formation of micro-sized erythrocyte ghosts (µEGs). µEGs were then sonicated (top sonicator FB705, Fisher Scientific, Hampton, NH, USA) to form nano-sized erythrocyte ghosts (nEGs) for further functionalization with folate. Specifically, we used 5 mg/mL of DSPE-PEG (2000 Da)-folate (Nanosoft Polymers, Winston-Salem, NC, USA), grafted through lipid insertion to functionalize the nEGs with folate. We note that the commercially available folate is in the form of folic acid. To make F-IDNETs, 10 mL of folate-functionalized nano-EGs was first concentrated down into 1 mL of isotonic (1X PBS) by centrifugation at ~77,000× *g* at 4 °C for 1 h. We then added the 1 mL of the isotonic PBC containing the concentrated folate-functionalized nano-EGs to 3 mL of ICG (MP Biomedicals, Santa Ana, CA, USA) aqueous solution, 3 mL of Sorenson’s buffer, and 3 mL of DOX (VWR International, Radnor, PA, USA). The respective amounts of ICG and DOX were 6 and 3 mg, resulting in concentrations of 774 and 517 µM in the loading buffer, respectively. Samples were then centrifuged at 77,000× *g* at 4 °C for 1 h. Non-functionalized nanoparticles loaded with ICG alone (i.e., NETs), or with ICG and DOX (i.e., IDNETs), and folate-functionalized nanoparticles loaded with ICG but without DOX (i.e., F-NETs) were fabricated in a similar fashion as described above. Non-encapsulated ICG, DOX, and ICG + DOX dissolved in isotonic PBS at specified concentrations were used as additional control samples.

### 4.2. Physical and Optical Characterizations

The zeta potentials and hydrodynamic diameters of the various sets of nanoparticles were measured by DLS (Zetasizer Nano ZS90, Malvern Instruments Ltd., Westborough, MA, USA). Absorption spectra were obtained using a spectrophotometer (Jasco-V670 UV-vis spectrophotometer, JASCO, Easton, MD, USA) with optical path length of 1 cm. Fluorescence emission spectra of ICG-containing nanoparticles in response to 720 ± 2.5 nm excitation light, spectrally filtered from a 450 W xenon lamp, were recorded in the range of 735–900 nm using a fluorimeter (Fluorolog-3 spectrofluorometer, Horiba Jobin Yvon, Edison, NJ, USA). Fluorescence emission from DOX in response to photoexcitation at 470 ± 2.5 nm was recorded in the spectral range of 485–700 nm. All spectral recordings were made at room temperature.

### 4.3. Radiometric Temperature Measurements

**Figure 8:**
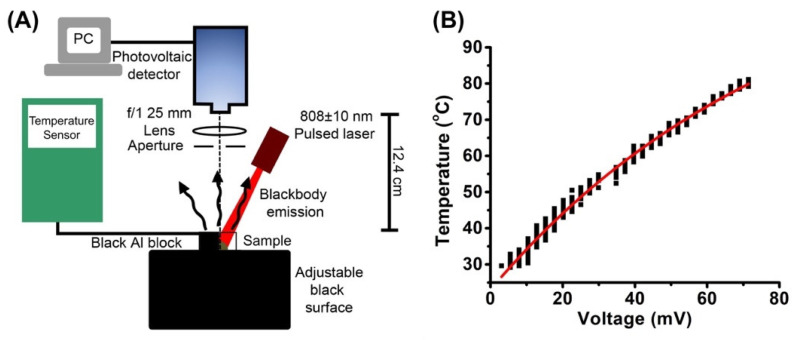
Radiometric temperature measurements. (**A**) Experimental setup for calibration and temperature measurements. Further details are provided in the methods sections. (**B**) Illustrative calibration curve relating radiometric surface temperature to the output voltage of the detector. Red trace represents the mathematical fit to experimental measurements.

We used infrared radiometry as a non-contact method for temperature measurements (Figure 8). Blackbody emission in the range 2–7 μm was detected by a ZnSe photovoltaic detector (PVD) (PVI-4TE-6,Vigo Systems, Poland) with 25 mm f/1 lens (Edmund Optics, 69649, BBAR 3–5 μm) placed 25 mm from detector. The PVD was connected to a data acquisition board (IOtech Systems, Wavebook 512, Newcastle upon Tyne, UK), and data were acquired using the WaveView software. A 5 mm aperture was used during calibration to match a 5 mm laser spot size for pulsed laser irradiations. To calibrate the output voltage of the PVD for temperature, an aluminum (Al) block painted black with black India Higgins ink (Chartpak, Leeds, MA, USA) was used to simulate a blackbody. The Al block was heated on a hotplate and placed on the measurement stage positioned 12.4 cm from detector. Infrared emission from the Al block during the cooling phase was recorded, while, simultaneously, a temperature sensor (HYPO-33-1-K-G-60-SMPW-M, Omega Engineering, Stamford, CT, USA) adherent to the surface and connected to a readout device (Vernier LabQuest), provided the temperature measurements (Figure 8A). Output voltage of the PVD and the measured surface temperature were plotted in MatLab (Mathworks), and the data were fitted mathematically to obtain a calibration curve (Figure 8B). For experimental temperature measurements, the sample or the animals were placed on the measurement stage and positioned 12.4 cm from the detector.

### 4.4. Pulsed Laser Irradiation

We used a pulsed 808 ± 10 nm laser (Lumics LuOcean Mini 4) coupled to a 10-mm aperture collimator (SMA 88170, Edmund Optics, NJ, USA) for laser irradiation experiments. Laser pulse duration (∆*τ*_laser_) was 500 ms. Laser energy was determined using an energy meter (Ophir 3(150)-HE-SH, Ophir Optronics, Jerusalem, Israel). The laser spot size laser was 5 mm in all experiments.

### 4.5. Photothermal Response and Photostability of Nanoparticles

Samples of Free ICG, NETs, F-NETs, IDNETs, and F-IDETs were irradiated at a laser fluence (*D*_o_) (radiant exposure) of 50 or 90 J/cm^2^ to assess the photothermal response and optical stability of ICG upon pulsed laser irradiation. After irradiation, we acquired the absorption spectra of the samples and quantified the photostability of ICG at 808 nm by normalizing its absorbance at 808 (A_808_) after laser irradiation to the 808 nm absorbance in the absence of laser irradiation (i.e., dark absorbance).

### 4.6. Effect of pH and Pulsed Laser Irradiation on DOX Release

To investigate the effects of pH and laser-triggered release of DOX, IDNETs and F-IDNETs were diluted in PBS (pH 5.5 or pH 7.6) and irradiated using *D*_o_ = 25 J/cm^2^. A total of 50 μL of irradiated sample was diluted with 450 μL of PBS and filtered using Amicon Ultra-4 filter tubes (EMD Millipore, Burlington, MA, USA) at 4k rpm for 10 min. For dark controls, particles were diluted to A_808_ = 1 in 1X PBS (at pH 5.5 or pH 7.6) and 500 μL was filtered using Amicon Ultra-4 filter tubes. After filtration, 200 μL of the supernatant was collected into 96-well plate. Additionally, the pellet was resuspended to the original volume of 500 μL, and 200 μL of sample was collected into a 96-well plate. DOX peak fluorescence emission at 595 nm, in response to 470 nm excitation, was recorded using a plate reader (Molecular Devices SpectraMax M3, San Jose, CA, USA). A percentage of DOX release was quantified as:

(2) $$\%\;\mathrm{DOX}\;\mathrm{Release}=\frac{Fl_{s}}{Fl_{s}+Fl_{p}},$$

where *Fl_s_* and *Fl_p_* represent the fluorescence of the supernatant and the pellet, respectively.

### 4.7. Cell Culture

SKOV3 ovarian cancer cells (ATCC, Manassas, VA, USA) were used for in vitro experiments and tumor implantation in Nu/J female nude mice. Cells were cultured in Rosewell Park Memorial Institute (RPMI) 1640 medium (Mediatech Inc., Manassas, VA, USA), supplemented with 10% fetal bovine serum (FBS) and 1% penicillin/streptomycin (Corning Inc., Corning, NY, USA) at 37 °C in 5% humidified CO_2_.

### 4.8. Fluorescence Imaging of Cancer Cells

Approximately 4 × 10^5^ SKOV3 cells in 200 μL of RPMI 1640 medium supplemented with 10% FBS and 1% Penicillin/Streptomycin were added to each well of a 96-well plate. Cell suspensions were stored in 5% CO_2_ overnight. All samples were diluted to have same absorbance at 808 nm. On the following day, the cells were washed and incubated with various agents consisting of 1X PBS, free ICG, free DOX, free ICG + DOX, NETs, IDNETs, F-NETs, or F-IDNETs in separate wells for 4 h in the dark at 37 °C. After incubation, cells were washed twice with 1X PBS and fixed using 4% paraformaldehyde (Electron Microscopy Sciences, Hatfield, PA, USA), permeabilized with 2% Tween-20 (Sigma Aldrich, St. Louis, MO, USA), and finally incubated with 300 nM 4′,6-diamidino-2-phenylindole (DAPI) for 5 min to stain the nuclei for fluorescence imaging. NIR fluorescence emission (>770 nm), in response to 740 ± 30 nm excitation light from a Nikon halogen lamp, was captured by an electron multiplying charged-coupled device (EM-CCD) camera (Quant EM-CCD, C9100-14 Hamamatsu, Shizuoka-ken, Japan). The camera exposure time was set at 0.1 s. Fluorescence emission from DOX in the range 524 ± 24 nm was collected in response to the 485 ± 35 nm excitation light, filtered from the halogen lamp. Fluorescence emission from DAPI-stained nuclei in the range of 435–485 nm was collected in response to 360 ± 20 nm excitation, filtered from the Nikon halogen lamp. We present falsely coloured microscopic fluorescence images associated with NIR emission due to ICG (red channel), DOX emission (green channel), and visible emission due to DAPI (blue channel).

### 4.9. Cell Viability Assay

SKOV3 cells incubated with 1X PBS and various agents were plated in a 24-well plate in triplicate. Dark controls were subject to treatment with various agents, but without laser irradiation. Plated SKOV3 cells were left overnight. On the following day, SKOV3 cells were washed with 1X PBS and incubated with 250 μL RPMI 1640, supplemented with 10% FBS and 1% Penicillin/Streptomycin. In total, 250 μL of cell samples were incubated for 4 h at 37 °C, supplemented with 5% CO_2_, and then washed twice with 1XPBS and trypsinized. For laser irradiation experiments, cells were concentrated into 50 μL samples. After irradiation (*D*_o_ = 50 or 90 J/cm^2^, ∆*τ*_laser_ = 500 ms), cells were returned to a plate and incubated overnight at 37 °C, in the presence of 5% CO_2_. On the following day, 3-(4,5-dimethylthiazol-2-yl)-2,5-diphenyl tetrazolium bromide (MTT) reagent (Sigma Aldrich, St. Louis, MO, USA) was added. After 4 h of incubation at 37 °C, in the presence of 5% CO_2_, the solution was aspirated and formazan crystals were dissolved in 100% dimethyl sulfoxide (Corning Inc., Corning, NY, USA). Absorbance was measured at 572 nm using the SpectraMax M3 plate reader. Cell viability was then determined using a calibration curve obtained from SKOV3 cells in a 96-well plate.

### 4.10. Animal Studies

Female Nu/J mice (20–25 g, 6–8 weeks) were purchased from Jackson Laboratory (Bar Harbor, ME, USA). We injected ~1 × 10^7^ SKOV3 cancer cells subcutaneously into the thighs. Mice were monitored until the tumor sizes reached approximately 15 mm^3^. The tumor volume was calculated as *D* × *d*^2^/2, where *D* and *d* were the larger and smaller diameters of each tumor.

Tumor-bearing mice were randomly divided into six groups with six animals in each group (Table 1). The administered dose of ICG was within the 2–4 mg/kg range used in humans [42,43]. The administered doses of ICG and DOX were also much lower than the respective LD_50_ values of 62 mg/kg [44] and 10 mg/kg [45] in mice. We administered 100 μL of each of the agents intravenously via the tail vein while the animal was anesthetized. Pulsed (0.5 s) 808 nm laser irradiation was performed at 24 h post-injection using *D_o_* = 90 J/cm^2^ at 5 mm spot size.

We measured the radiometric surface temperature change in response to pulsed 808 nm laser irradiation using the calibrated PVD. Following each treatment, animals were allowed to recover. We assessed the effectiveness of the different administered agents in mediating tumor destruction by measuring the tumor volumes for up to 14 days following each treatment. We estimated the relative tumor volumes (V/V_o_) during this time interval by dividing the volume of each tumor (V) on the measurement day by the initial tumor volume (V_o_) on the day of sample injection. All the animals were subsequently euthanized on day 14. The body weight of mice were monitored every 1–2 days for 2 weeks after sample injection.

### 4.11. Caspase-3 Staining

A subset of mice from each group was euthanized 24 h post laser irradiation for histological analysis. Tumors were extracted and sectioned for staining with FITC-labeled caspase-3 antibody (BD Biosciences, San Diego, CA, USA) to assess apoptosis. Fluorescence emission (524 ± 24 nm) in response to 485 ± 35 nm excitation, filtered from a Nikon Mercury/Xenon arc lamp, was captured by the EM-CCD camera with exposure time set at 0.1 s. Mean and SDs of the image intensities (n = 3 images) were quantified using ImageJ.

## 5. Conclusions

We have engineered nanoparticles derived from erythrocytes that contain the photothermal agent ICG and the chemotherapeutic drug DOX. The surface of the nanoparticles is functionalized with folate. Our in vitro and in vivo animal model studies indicate that the dual treatment modality based on photothermal heating and photo-induced release of DOX combined with folate targeting can provide a more effective approach in killing the cancer cells, as compared to single treatment modality using photothermal heating or chemotherapy.

## Figures and Tables

**Table 1:** Description of the groups for the animal studies.

Group Number	Administered Agent (Dose)	Laser Irradiation (Yes/No)
Group 1	PBS	Yes
Group 2	Free ICG (2.67 mg/kg)	Yes
Group 3	Free DOX (0.76 mg/kg)	No
Group 4	Free ICG (2.67 mg/kg) + Free DOX (0.76 mg/kg)	Yes
Group 5	F-NETs (2.67 mg ICG/kg)	Yes
Group 6	F-IDNETs (2.67 mg ICG/kg + 0.76 mg DOX/kg)	Yes

## Data Availability

Not applicable.

## Abbreviations

**Abbreviation**	**Definition**
A_808_	Absorbance at 808 nm
Al	Aluminum
CW	Continuous wave
DAPI	4′,6-diamidino-2-phenylindole
DLS	Dynamic light scattering
DOX	Doxorubicin hydrochloride
DSPE	1,2-distearoyl-sn-glycero-3-phosphoethanolamine
EM-CCD	Electron multiplying charged-coupled device
F-IDNETs	Folate-functionalized ICG + DOX containing NETs
F-NETs	Folate-functionalized NETs
FBS	Fetal bovine serum
FDA	Food and Drug Administration
FITC	Fluorescein isothiocyanate
FR-α	Folate receptor-α
ICG	Indocyanine green
i.v.	Intravenous
IDNETs	ICG + DOX containing NETs
LD50	Lethal dose that causes death in 50% of animals
nEGs	nano-sized erythrocyte ghosts
NETs	NIR erythrocyte-derived transducers
PBS	Phosphate buffer saline
PTT	Photothermal therapy
PVD	Photovoltaic detector
RBCs	Red blood cells
RPMI	Rosewell Park Memorial Institute
SD	Standard deviation
UV-Vis	Ultraviolet-Visible
µEGs	Micro-sized erythrocyte ghosts
